# Nephrectomy for xanthogranulomatous pyelonephritis—a not-so-simple solution

**DOI:** 10.1007/s11845-023-03496-2

**Published:** 2023-08-23

**Authors:** Caroline Kelly, Steven Anderson, Aisling Looney, Naomi Shannon, Radha Senaratne, Eabhann O’Connor, Kieran Breen, Gerald Lennon, Barry McGuire, Michael Murphy, Diarmaid Moran, David Galvin

**Affiliations:** Urology Department, St. Vincent’s University Hospital, Dublin, Ireland

**Keywords:** Adult, Nephrectomy, Pyelonephritis, Urinary tract infection, Xanthogranulomatous pyelonephritis

## Abstract

**Background:**

Xanthogranulomatous pyelonephritis (XGP) is a rare chronic inflammatory condition of the kidney, associated with high patient morbidity, often requiring targeted antibiotic therapy and surgical removal of the affected kidney.

**Aim:**

We report the outcomes of patients undergoing nephrectomy for XGP in our institution over a 12-year period.

**Methods:**

Following ethical approval, a retrospective review of histological samples of renal tissue demonstrating features of XGP from June 2010 to 2022 was conducted. Laboratory, imaging, and clinical data of included participants were collected.

**Results:**

Eleven patients were included (8 women, 3 men), mean age of 58.1 (35–81). Recurrent urinary tract infection was the most common clinical presentation (55%, *n* = 6). Other presentations included flank pain (36%, *n* = 4), collection/ abscess (45%, *n* = 5), and nephro-cutaneous fistulae (9%, *n* = 1). The majority of patients had bacteriuria (91%, *n* = 10), and *Escherichia coli* was the most common bacteria isolated (55%, *n* = 6). Antibiotic resistance was seen in 60% of positive urine samples (*n* = 6). An open nephrectomy was performed in all but one case (91%, *n* = 10). A postoperative complication occurred in 73% (*n* = 8), with 50% (*n* = 4) of complications Clavien Dindo grade 3 or higher, including one patient mortality.

**Conclusions:**

XGP is a difficult and complex condition to treat. All patients in this series presented with infection or associated sequelae thereof. Complex XGP cases therefore often require open nephrectomy and have high rates of postoperative complications. Careful consideration of antibiotic and operative intervention is therefore essential to ensure the best outcome for these patients.

## Introduction

Xanthogranulomatous pyelonephritis (XGP) is a rare form of chronic pyelonephritis first described by Schlagenhaufer et al. in 1916. Macroscopic features characteristic of the disease include an enlarged kidney with perirenal fibrosis and extension of the inflammatory process into the retroperitoneal space [1]. Microscopically, the normal renal parenchymal cells undergo necrosis with phagocytosis of lipids by macrophages. This results in the characteristic appearance of foamy xanthomatous histiocytes [1]. There is a female preponderance and XGP can occur at any age. Complications of this condition can be life-threatening with many patients requiring admission to the intensive care unit [2, 3]. Common presentations include flank pain, recurrent urinary tract infection (UTI), and a perirenal collection or mass [4]. There is no recognised guideline outlining the correct management of XGP, likely due to the heterogeneity of the condition; however, treatment with antimicrobial therapy and surgical removal of the affected kidney is widely regarded as the mainstay of treatment [5, 6]. *Escherichia coli* (*E. coli*) and *Proteus mirabilis* are the most commonly cultured pathogens identified in urine samples taken from patients diagnosed with XGP, with varying degrees of antimicrobial resistance reported [5–7]. This disease process can often mimic other renal pathologies and can therefore be difficult to diagnose and treat. Cross-sectional imaging with computed tomography (CT) is regarded as the most accurate diagnostic tool to diagnose XGP [8]. The aim of this review is to summarise laboratory, radiological, and operative findings of patients diagnosed and treated with xanthogranulomatous pyelonephritis in our centre over a 12-year period, to guide future antimicrobial and surgical management.

## Methods

Following ethical approval from the research and ethics committee at our institution, a retrospective review was undertaken (ethical IRB number RCR22-022). All cases of XGP managed by surgical excision between June 2010 and 2022 were included. Cases were identified from the Department of Pathology database. A search of renal tissue specimens was conducted using the terms “xanthogranulomatous pyelonephritis” and “acute or chronic inflammation”. The diagnosis of XGP was confirmed by histological evidence of replacement of the renal parenchyma with foamy histiocytes. Data collected from medical records included age, gender, medical history of diabetes mellitus or compromised immune system, presenting complaint, prior abdominal imaging including computed tomography (CT) and renogram studies, presence of renal/ ureteric calculi, operative intervention performed, and intraoperative and postoperative complications. Urine culture results were analysed to identify bacterial growth and antibiotic resistance. A descriptive analysis of the results was performed.

## Results

### Patient demographics

A total of 11 patients underwent nephrectomy for XGP during the study period. The majority of patients were female (73%, *n* = 8). All cases were unilateral with 7 cases involving the left kidney and 4 involving the right. The patient demographics are summarised in Table 1.

**Table 1:** Demographics

*Variable*	*Value*
Female vs male	8 (73%) vs 3 (27%)
Age in years (mean)	58 (range 35–81)
Side	
Right	4 (36%)
Left	7 (64%)
Diabetes mellitus	2 (18%)
Chronic liver disease	1 (9%)
Nephrolithiasis	
Staghorn calculus	10 (91%)
Hydronephrosis	2 (18%)
Clinical presentation^a^	
Collection/abscess	5 (45%)
Recurrent UTI^b^	6 (54%)
Urosepsis	5 (45%)
Flank pain	4 (36%)
Nephro-cutaneous Fistula	1 (9%)

^a^Patients presented with more than one presenting complaint

^b^Urinary tract infection

### Clinical features

**Fig. 1:**
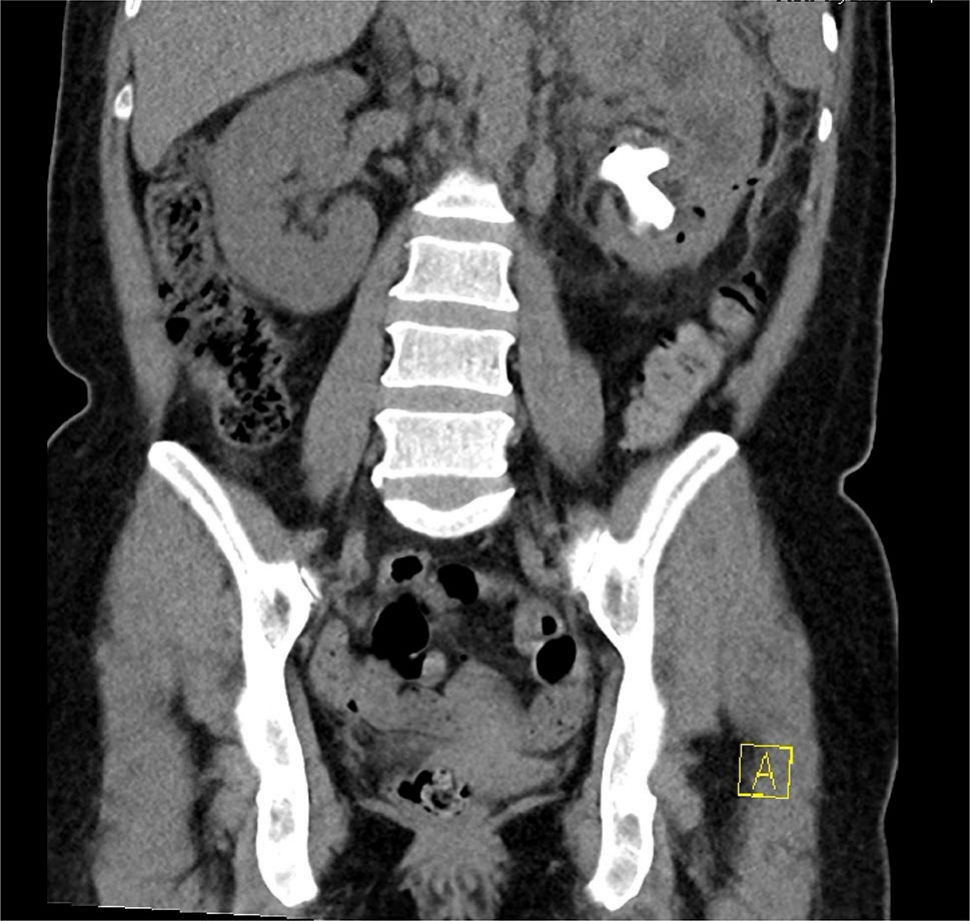
Computed tomography scan of the abdomen and pelvis showing emphysematous pyelonephritis of the left kidney with marked hydronephrosis. Partial staghorn calculus left kidney

A positive mid-stream urine culture was seen in 91% (*n* = 10) of cases, with *E. coli* (60%, *n* = 6) the most frequently cultured pathogen. Antimicrobial resistance was a common feature, identified in 55% (*n* = 6) of cases. All patients underwent cross-sectional imaging in the form of a computed tomography scan. Urolithiasis was seen in all cases, and an obstructing staghorn calculus was seen in 91% (*n* = 10) of cases. Typical CT features noted were perinephric stranding and cortical thinning in the affected kidney. A peri-nephric collection or abscess was identified on CT in 45% of cases (*n* = 5), with one case further complicated by a nephro-cutaneous fistula. Figure 1 demonstrates an oedematous, hydronephrotic left kidney with a partial staghorn calculus in a patient presenting with acute emphysematous pyelonephritis, subsequently diagnosed with XGP. A preoperative renogram was performed in 8 (73%) cases. The mean contribution of the affected kidney to the overall split-function was just 13% (range 0–36%). The microbiological and radiological findings are summarised in Table 2.

**Table 2:** Microbiological and radiological findings

*Variable*	*Value*
Positive urine culture	10 (91%)
*E. coli*^a^	3
*Enterococcus faecalis*	1
*Proteus mirabilis*	1
*Streptococcus anginosis*	1
*Mixed*	
* E. coli* + *Enterococcus faecalis*	2
* E. coli* + *Proteus mirabilis*	1
* Serratia marcescens* + *Enterococcus* species	1
Resistance profiles	
Quinolones	0
Carbapenems	0
Amoxicillin/clavulanic acid	3
Piperacillin/tazobactam (TZP)	1
Ceftriaxone	1
Fosfomycin	0
Trimethoprim/sulfamethoxazole(TMP/SMX)	0
Aminoglycoside	1
Renogram pre nephrectomy	
DTPA (mean % function XGP kidney)	2 (0%)
DMSA (mean % function XGP kidney)	6 (17%) (range 0–36%)
CT findings pre nephrectomy Malek & Elder classification	
1	3
2	0
3	8

^a^*Escherichia coli*

### Operative outcomes

**Fig. 2:**
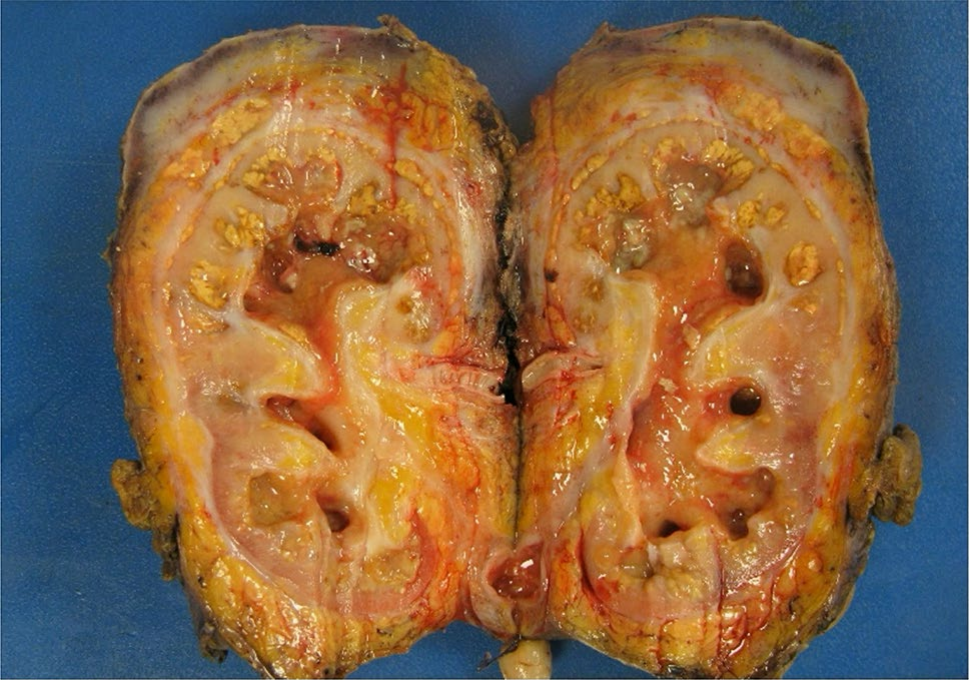
Macroscopic appearance of XGP kidney

All patients underwent surgical removal of the affected kidney. Figure 2 demonstrates the macroscopic appearance of an XGP kidney once removed. The majority of cases (82%, *n* = 9) were performed electively for treatment of symptomatic XGP. Emergency nephrectomy was performed for two patients, one case presenting with urosepsis in an obstructed kidney and the second with renal abscess requiring drainage. Total number of days in hospital prior to and post nephrectomy was 27 days and 39 days, respectively. Elective cases were admitted for a mean of 21 days (range 2–37 days) with complications of XGP prior to elective admission for nephrectomy. Duration of admission for elective nephrectomy was an additional 22 days on average (range 8–35 days). Overall, mean time from initial presentation to nephrectomy was 172 days (range 41–491 days). An open approach was used in 91% (*n* = 10) of patients undergoing simple nephrectomy, with one patient undergoing a laparoscopic nephrectomy. This patient was a 77-year-old woman who presented with a small perirenal abscess on preoperative imaging with two fistulous tracts, one extending to the lateral abdominal wall and the second to the skin. This patient’s admission was complicated by a postoperative pneumoniae treated with intravenous antibiotics but they were successfully discharged 11 days post laparoscopic nephrectomy.

**Fig. 3:**
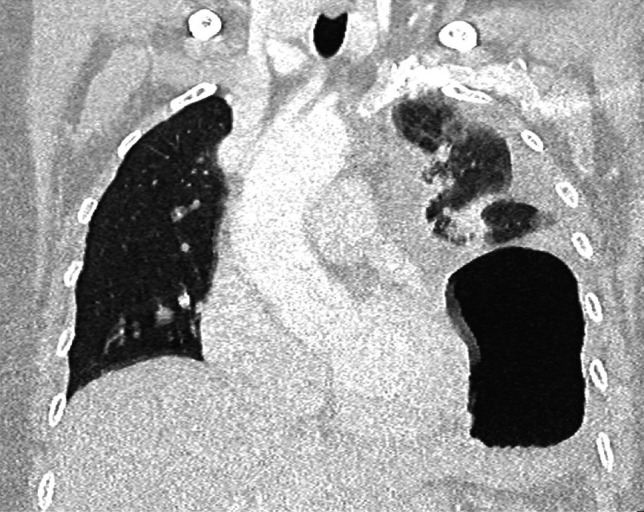
Computed tomography scan of chest demonstrating left diaphragmatic hernia

Postoperative complications were common, occurring in 73% (*n* = 8) of cases, highlighting the technical complexity in the management of these cases. Overall, four patients had a surgical site infection or pneumonia requiring intravenous antibiotics (Clavien Dindo 1), while a further four patients had higher grade complications (≥ Clavien Dindo 3). Two patients had a postoperative collection requiring insertion of a drain under sedation (Clavien Dindo 3a). One patient suffered a diaphragmatic injury which was identified and repaired intraoperatively. Subsequently, this patient developed a diaphragmatic hernia, demonstrated in Fig. 3, requiring repair under general anaesthetic (Clavien Dindo 3b). There was one postoperative mortality. This patient was an 81-year-old male with a history of chronic liver disease and type 2 diabetes mellitus who re-presented 22 days post-operatively with a collection at the nephrectomy bed and died 9 days following readmission despite drainage of postoperative collection and targeted antibiotic therapy. The operative outcomes are summarised in Table 3.

**Table 3:** Nephrectomy data and complications

Nephrectomy approach	
Open	10
Laparoscopic	1
Elective vs. urgent	9 (82%) vs 2(18%)
Intraoperative complications	
Diaphragmatic injury	1
Postoperative complications Clavien-Dindo classification	
1	4
2	0
3a	2
3b	1
4	0
5	1

## Discussion

XGP is a rare chronic inflammatory disease of the renal parenchyma caused by chronic infection and obstruction. Similar to prior case series [9], XGP was noted to affect women more than men in this series. Women are more frequently diagnosed with urinary tract infections and are therefore hypothesized to be more predisposed to the complications of chronic pyelonephritis than men [9]. As identified in prior series, the left kidney was noted to be more commonly affected than the right with no clear cause identified [6, 8, 10].

The pathogenesis of XGP is associated with several aetiological factors including chronic urinary tract infection, renal obstruction, and the presence of renal calculi [9, 10]. Our series identified recurrent urinary tract infection in over 50% of cases, with evidence of obstruction identified in 18.2% of cases. Presence of staghorn calculi have been observed in the vast majority of patients with XGP [11]. This was also observed in this series with staghorn calculi identified in over 90% of cases.

There is limited antimicrobial guidance available in the literature on the treatment of XGP. The most frequent bacteria isolated from urine samples in XGP patients, based on published retrospective case series, include *Proteus mirabilis* and *E. coli* [5, 7]. Artiles-Medina et al. published a retrospective series of 27 patients diagnosed with XGP and reported rates of a positive urine culture of 48.1% with highest reported antibiotic resistance rates of 33.3% to fosfomycin and trimethoprim/sulfamethoxazole. Resistance to quinolones, amoxicillin/clavulanic acid, piperacillin/tazobactam, and ceftriaxone was also noted at reduced rates [5]. This series noted higher rates of positive urine cultures than previous series, with over 90% of cases demonstrating bacterial growth. Similar to prior case series, *E. coli* was the most common bacteria noted. Mixed infection was detected in 4 cases. Amoxicillin/clavulanic acid demonstrated the highest rates of resistance, with 30% of bacteria isolated demonstrating resistance. Resistance to piperacillin/tazobactam, ceftriaxone, and aminoglycosides was also noted.

The correct diagnosis of XGP can be challenging due to its ability to mimic other pathological conditions of the kidney. High-resolution imaging techniques have enhanced the diagnostic accuracy of this condition. Eastham et al. [8] retrospectively reviewed radiological records of 27 patients with a diagnosis of XGP and reported CT imaging correctly diagnosed XGP in 87% of cases. Zorzos et al. [2] retrospectively reviewed the CT imaging of 39 patients diagnosed with XGP and determined characteristic findings on CT imaging include a large calculus identified in the renal collecting system, a poorly functioning kidney with absent excretion of contrast, large lesions with poorly defined borders, and perirenal extension such as fat stranding, thickening of gerota’s fascia, or perirenal collection. All patients in this series had a preoperative CT abdomen pelvis in conjunction with a preoperative renogram in 73% (*n* = 8) of cases. Over 90% of cases had a staghorn calculus identified in the collecting system of the affected kidney. Of those cases who had a preoperative renogram, mean function of the affected kidney was 13% corresponding to a poorly functioning kidney. Similar to previous data published, this series identified perirenal disease on preoperative imaging in the majority of cases (73%, *n* = 8).

In 1978, Malek and Elder subdivided the disease process of XGP into 3 stages according to the extent of disease involvement of the kidney and surrounding structures. Stage 1 describes disease confined to the kidney. Stage 2 describes disease involving renal parenchyma extending into perinephric fat. Stage 3 describes disease extending into the retroperitoneal space involving adjacent structures [1]. The majority of cases in this series were diagnosed with stage 3 disease on preoperative imaging. “Simple nephrectomy” for XGP, regardless of stage of disease, remains technically challenging due to the presence of extensive adhesions and the frequent involvement of local structures, particularly in stage 3 disease. Flynn et al. [12] reviewed the operative management of 27 cases of XGP and encountered deep invasion into psoas muscle in 5 cases, invasion into diaphragm and aorta in one case, and bowel involvement in 4 cases with 2 cases requiring hemicolectomy. Extensive adhesions and invasion of local structures mean the majority of nephrectomies for XGP are performed open. This was reflected in the current series with over 90% of cases performed open. One case demonstrated invasion of the diaphragm resulting in diaphragmatic injury and subsequent hernia requiring repair. The use of laparoscopy in the surgical treatment of XGP is uncommon with a considerable amount of experience required [13]. In this series, one case was successfully performed laparoscopically. Overall, in this series, the postoperative complication rate was 73% (*n* = 8) with 36% (*n* = 4) of patients having a Clavien Dindo complication of grade 3 or higher. This is slightly higher than previously published case series, with Artiles-Medina et al. [5] reporting 27% (*n* = 7) of 27 cases in their series experiencing a Clavien Dindo complication of grade 3 or higher.

## Conclusion

XGP is a rare complication of chronic pyelonephritis that is associated with a high rate of morbidity and mortality despite surgical management. The ability to recognise characteristic features on cross-sectional imaging is vital in obtaining a prompt diagnosis. There are multiple causative microorganisms with variable resistance patterns described, and as such necessitate careful consideration of antimicrobial treatment. Despite appropriate antimicrobial treatment, surgical management, though technically challenging, remains necessary in the majority of cases and is associated with a high rate of postoperative complications.

## Data Availability

The authors confirm that the data supporting the findings of this study are available within the article.
